# A Naturalistic Prospective Study of the Prognostic Impact of EPHX2 in Major Depressive Disorder: Impulsivity may be an Important Factor

**DOI:** 10.1155/da/8124403

**Published:** 2025-04-13

**Authors:** Shuqiong Zheng, Jia Guo, Rongxin Zheng, Yujia Ji, Quan Zhong, Honglei Yin

**Affiliations:** ^1^Department of Psychiatry, The Third Affiliated Hospital, Southern Medical University, Guangzhou, China; ^2^Department of Biostatistics, Columbia University, New York, New York, USA

**Keywords:** cognitive function, EPHX2, impulsivity, major depressive disorder, suicide

## Abstract

**Background:** Major depressive disorder (MDD) is a leading cause of disability worldwide. The pathophysiology of MDD remains unclear, which limits the development of treatments for MDD. Recently, epoxide hydrolase 2 (EPHX2) has been found to be associated with MDD. Our previous study revealed an association between EPHX2 expression and suicide. However, the effect of EPHX2 on the prognosis of MDD and suicide remains unclear. Previous studies have found that impulsivity at baseline can be a significant predictor of clinical improvement in patients with MDD. Therefore, we inferred that EPHX2 could be associated with the treatment effect of MDD, and impulsivity could mediate the effect of EPHX2 on the treatment effect of MDD.

**Methods:** This naturalistic prospective study included 117 participants with MDD, who were assessed, using clinical questionnaires, cognitive function, and treatment information, at baseline, 2 weeks, and 1, 2, and 3 months. A linear mixed-effects model was used to investigate longitudinal changes in the severity of symptoms, risk of suicide, and cognitive function.

**Results:** The interactive effects of impulsivity and EPHX2 polymorphisms on the risk of suicide (measured by the Columbia-Suicide Severity Rating Scale) were significantly different for rs11288636, rs68012435, and rs11288636. The interactive effects between polymorphisms and time on depression severity (measured by the Hamilton Depression Scale-24) were significantly different and including after adjustment for the total impulsivity score.

**Conclusions:** This study suggests that EPHX2 polymorphisms are associated with the prognosis of MDD, and impulsivity could be a critical factor for the change in suicide risk among different EPHX2 genotypes.

**Trial Registration:** ClinicalTrials.gov identifier: NCT05575713

## 1. Introduction

Major depressive disorder (MDD) is a leading cause of disability worldwide, with significant socioeconomic implications [1]. As the major cause of suicide-related years of life loss, MDD increases the risk of death by suicide twentyfold [2]. The treatment response to selective serotonin reuptake inhibitors, typical antidepressants for MDD, is 40%–70% [3]. Due to the complex and multifaceted influence of both genetic and environmental factors, the biological mechanisms underlying MDD remain unclear [4, 5]. Currently, accumulating evidence suggests the involvement of neuroinflammation in the pathophysiology and treatment of MDD [6–8].

The human *EPHX2* (epoxide hydrolase 2) gene codes for the soluble epoxide hydrolase (sEH) protein is widely expressed in a number of tissues. The sEH protein primarily catalyzes the hydrolysis of epoxides, such as epoxyeicosatrienoic acids (EETs), into their corresponding diols. These epoxides and diols play important roles in inflammatory responses and metabolic processes. Given the key role of inflammation in MDD, sEH has recently been considered a therapeutic target for neuropsychiatric disorders, including MDD [9].

Cao et al. were the first to find that the deletion of *EPHX2* could lead to the antidepressant-like effects in animal models of depression [10]. Higher sEH protein levels were found in both patients with MDD and rodents with depression-like phenotypes [11]. In addition, rapid antidepressant-like effects for sEH inhibitors were confirmed in multiple animal models of depression [9, 10]. Thus, sEH inhibitors are regarded as potential therapeutic or prophylactic drugs for MDD. A series of studies have further explored the mechanisms underlying the antidepressant-like effects of *EPHX2*, including astrocytic ATP releasing, brain–liver axis, and brain–gut–microbiota axis [9, 10, 12, 13]. However, the role of *EPHX2* polymorphisms in the treatment effect in MDD patients remains unknown.

Cognitive impairment has recently been regarded as a core feature of patients with MDD [14]. Decreased cognitive performance has been reported in multiple domains, including memory and attention [15–17]. However, current therapies for MDD, including psychotherapy and antidepressant medications, barely address cognitive symptoms [18]. Previous studies have found that the deletion of *Ephx2* and sEH inhibitor could ameliorate cognitive declines in Alzheimer's disease (AD) mouse models [19, 20]. Besides, the immunoreactivity of sEH was associated with age-related vascular cognitive impairment (VCI) [21]. The sEH inhibitor may represent a viable target for the treatment of cognitive impairment in AD and VCI [22, 23]. Though we did not found a direct effect between the mRNA expression level of *EPHX2* and MDD, a significant indirect effect was found through cognitive function between depression and the rs9331949 of *EPHX2* [24]. Thus, further study to explore whether *EPHX2* polymorphisms can affect changes in cognitive function is needed.

MDD is among the most commonly cited risk factors for suicidal thoughts and behaviors [25]. Our previous study found a significant association between suicide and a gene haploblock (rs9331942 and rs2279590) of *EPHX2* and subthreshold associations between eight single nucleotide polymorphisms (SNPs) of *EPHX2* and suicidal behavior [24]. MDD patients with allele C of rs2279590 may reverse the risk effect of allele G of rs9331942 on suicide. These findings suggest that *EPHX2* polymorphisms may influence the risk of suicide in MDD patients; however, whether they can affect changes in suicide risk during treatment remains unknown.

A previous study found that baseline impulsivity was a significant predictor of clinical improvement in patients with MDD [3]. Our previous study found that elevated cognitive impulsivity is a key factor for effective screening of patients with MDD [26]. Impulsivity is a distinct risk factor for suicide [27]. Heightened impulsivity may increase the severity of suicidal ideation in MDD [28, 29]. Monitoring and addressing impulsivity could aid in prevention and interventions for suicides in MDD [30]. To date, no study has explored the interactive function between *EPHX2* and impulsivity in the prognosis of MDD.

Therefore, we speculate that the *EPHX2* polymorphisms might be useful in the treatment of MDD. We compared changes in depressive symptoms, risk of suicide, and cognitive function after clinical treatment in patients with MDD and different *EPHX2* genotypes. We hypothesized that *EPHX2* could mediate the effect of impulsivity in the treatment of MDD.

## 2. Methods and Materials

### 2.1. Participants

A naturalistic prospective study was conducted to illustrate the longitudinal changes in symptoms of patients with MDD, who were psychiatric outpatients at Nanfang Hospital (Guangzhou, China) and recruited for this study between June 2021 and September 2022. Two experienced clinical psychiatrists interviewed all the patients. Cohen's kappa coefficient was used to assess diagnostic agreement between the two psychiatrists (kappa > 0.9). Demographic data (age, sex, educational level, and marital status) and clinical information (age at onset, episode duration, history of psychiatric medication use, and family history of psychiatric disorders) were collected. The patients were 18–65 years old.

The fifth edition of the Diagnostic and Statistical Manual of Mental Disorders (DSM-5) was used to ascertain the diagnosis of MDD. Patients with a score of >20 on the Hamilton Depression Scale-24 items (HAMD-24) (indicating currently depressed) were enrolled in the study [31]. Individuals who met any of the following criteria were excluded: (a) any history of psychiatric disorders, such as schizophrenia, bipolar disorder, or personality disorder; (b) using drugs including mood stabilizers, antidepressants, anxiolytics, antipsychotics, and benzodiazepines within the previous 2 weeks; (c) current unstable or serious somatic illness [32]; (d) current or any history of psychotic symptoms; or (e) lacking the capacity to provide informed consent.

**Figure 1:**
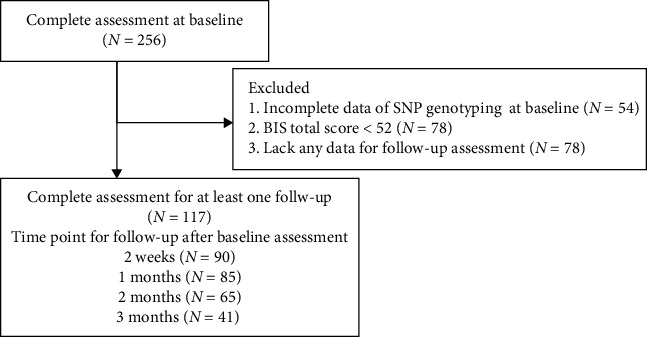
Flowchart.

After the baseline assessment, patients were followed for 3 months. During the 3-month follow-up period, patients were assessed at 2 weeks and 1, 2, and 3 months after the baseline assessment. Considering the impact of COVID-19, only patients who completed at least one follow-up assessment were included in the analysis. Based on the 3-month clinical follow-up, patients who developed manic or hypomanic symptoms—indicating a potential diagnosis of bipolar disorder—were excluded from the study (Figure 1).

This study was approved by the Ethics Review Committee of the Southern Hospital of Southern Medical University (approval number: NFEC-2022-092). All participants provided written consent before participating in the study.

### 2.2. Clinical Assessment

#### 2.2.1. HAMD-24

The HAMD-24 is a widely used rating scale to measure the severity of depressive symptoms [33]. It is based on the clinician's interview with the patient and probes for symptoms such as depressed mood, feelings of guilt, suicide, sleep disturbances, anxiety, and weight loss. Depressive symptom severity was assessed at baseline and at each follow-up visit. The scale includes 24 items, each scored based on the severity of symptoms, with scores ranging from 0 to 4 or 0 to 2 depending on the specific item. A higher total score indicates more severe depressive symptoms. The severity of depression was classified as follows: 0–7 points: no depressive symptoms; 8–20 points: mild depression; 21–35 points: moderate depression; 36 points and above: severe depression. In this study, we used a cutoff value of 20 points, thus including MDD patients scoring above 20.

#### 2.2.2. C-SSRS

Suicidal ideation and behavior within 30 days were measured using the C-SSRS, a semi-structured interview tool. The total C-SSRS score ranges from 0 to 42 [34]. Suicide risk was assessed at baseline and each follow-up visit.

#### 2.2.3. BIS-11

Impulsivity was measured using the BIS-11, which is a self-report questionnaire designed to assess the personality/behavioral construct of impulsiveness. The BIS-11 contains 30 items, with scores ranging from 0 to 4, evaluating three distinct dimensions of impulsivity: Cognitive Impulsiveness, Motor Impulsiveness, and Non-Planning Impulsiveness [35]. The Cognitive Impulsiveness subscale consists of items that assess a tendency to make decisions without sufficient thought, with scores ranging from 8 to 32 points. Higher scores on this subscale indicate a greater tendency for quick, unreflective decision-making. The motor impulsiveness subscale includes items that measure the tendency to act on impulse without considering the consequences, with scores ranging from 11 to 44 points. Higher scores reflect a stronger propensity for acting spontaneously. The nonplanning impulsiveness subscale consists of items that assess a lack of future-oriented thinking or planning, with scores ranging from 11 to 44 points. Higher scores on this subscale suggest a lower tendency to consider long-term consequences or plan ahead [36, 37].

Considering that this study is a preliminary exploration of the impact of impulsivity on the prognosis of MDD, only BIS total scores were used for group classification in this study. BIS-11 total scores of 52–71 were considered within the normal limits for impulsiveness (normal impulsivity, NI). Participants with a total score of ≥72 were regarded as highly impulsive (high impulsivity, HI). Seven participants with scores of ≤52 were excluded from subsequent statistical analysis because they were considered to have not completed the questionnaire honestly [36]. The impulsivity trait was assessed only at baseline.

### 2.3. Cognitive Function Assessment

We used three tasks to assess different domains of cognitive function: the Attention Network Test (ANT), the Suicide Stroop task (SST), and two versions of the *N*-back task (1-back and 2-back). All tasks were presented on a computer with a 24-inch screen. The participants were seated at a distance of 0.8 m from the screen. E-Prime 2.0: Professional SP1 (version 2.0.10.356, Psychology Software Tools, Inc. Pittsburgh, PA) software was used to present the stimuli for the task and to record the response time (RT) and accuracy (ACC). The participants were instructed to respond by pressing a button (F or J) on their keyboard as quickly and accurately as possible. All tasks were performed at baseline and follow-up visits.

The ANT was used to assess the efficiency of the three attention networks: alerting, orienting, and executive control (conflict) [38]. The mean RT and ACC were also calculated using the ANT. The SST has been used to assess executive function, processing speed, and attention bias [39]. The RT and ACC for neutral, negative, positive, and suicide-related words were calculated using the SST [39]. The 1-back and 2-back tasks were used to assess the working memory processing load. Mean RT and ACC were calculated for both tasks [40]. Detailed information regarding each task can be found in our previous study [24]. Seventeen features were acquired from three tasks (Table 1).

### 2.4. DDD of Antidepressant

The Anatomical Therapeutic Chemical (ATC) classification system and DDD are World Health Organization-recommended measuring units for drug utilization studies (http://www.whocc.no/atc_ddd_index/). The DDD is the assumed average maintenance dose per day, calculated from the dosage recommendations from each drug's product information [41]. Psychotropic medications were classified into three groups according to the ATC classification: antipsychotics (ATC code: N05A), antidepressants (ATC code: N06A), and benzodiazepine derivatives (ATC codes: N003AE, N05B, N05C). When patients received two or more drugs, the summed DDD was calculated for each category [42]. Finally, the DDD of antipsychotics, antidepressants, and benzodiazepines was calculated at each follow-up and regarded as a covariate in further analysis.

### 2.5. SNPs Genotyping of *EPHX2*

DNA was isolated from venous blood using the standard phenol-chloroform method. Eight SNPs in *EPHX2* associated with MDD and suicide attempts were included in this study [24]. These SNPs include rs11288636, rs68012435, rs7018249, rs7829267, rs56834178, rs9331942, rs17466684, and rs9331949. All eight SNPs are associated with a higher risk of suicide in MDD. Specifically, a significant association was identified between suicide and a gene haploblock (rs9331942 and rs2279590) of *EPHX2*. Subjects with haplotype GC and AT exhibited a lower rate of suicide attempts, suggesting that allele C of rs2279590 may reverse the risk effect of allele G of rs9331942 on suicide. Furthermore, SNP rs9331949 is associated with an increased risk of MDD, with its impact on depression potentially mediated through effects on cognitive function.

The genotyping was conducted by the BIOMIAO Company (Guangzhou Biomiao Biotechnology Co. Ltd.) by matrix-assisted laser desorption ionization time-of-flight mass spectrometry with a MassARRAY system (Agena Bioscience, Inc., USA). Information on SNP genotyping was assessed only at baseline. We coded 1 for individuals with minor allele, and 0 for those without minor allele (Table 1).

### 2.6. Statistical Analysis

Statistical analyses were performed using the SPSS (version 22.0; IBM Corp., Armonk, NY, USA) and R software (version 3.6.1 https://www.R-project.org/). Univariate analyses were performed using the Mann–Whitney *U* test and *t*-test for continuous variables based on whether the data conformed to a normal distribution. Data that conformed to the normal distribution are represented by mean ± standard deviation, while data that did not conform to the normal distribution is represented by median (quartile). Categorical variables were analyzed using the chi-squared test. Principal component analysis (PCA) was used to reduce the dimensions of cognitive function.

For the longitudinal change in the severity of the symptom, the risk of suicide, and the performance of the cognitive function, we used the linear mixed-effect model using R package “lme4” to investigate the effect of the group (the first step was for interaction between impulsivity and different genotyping of SNP, the second step was for different genotyping of SNP), time (the number of days between follow-up and baseline), and interaction (group × time), adjusted for age, sex, age at onset, age at current episode, educational level, recurrent episode or not, and the DDD of the antidepressant.

To assess the interactive effect, we defined four groups (N_0, N_1, H_0, and H_1) according to different types of impulsivity and SNPs. H for individuals with HI, and N for NI. H_1 means individuals with HI and minor allele of SNPs. When investigating the effect of different SNP genotypes, only significant SNPs in the first step were included, and the BIS-11 total score was a covariate in the second step. In the linear mixed-effect model, we put “subject” in the random effect, and “time,” “group,” and “time × group” in the fixed effect with a varying intercept. Statistical significance was set at *p* < 0.05. As there were eight SNPs, we used Bonferroni correction for multiple comparisons.

Due to the limited sample size, we conducted a power analysis of the linear mixed-effect model using the simr [43] package in R, which estimates power through Monte Carlo simulations for mixed-effect models. Our study aimed to estimate the additional changes in the C-SSRS total score over time for different groups of MDD patients, with H_0 (major allele carriers of rs11288636) as the reference group, at a significance level of 0.05. Thus, we calculated the power under different assumed effect sizes of the regression coefficients, including the value estimated using our data. Same as the main analysis, the model adjusted for gender, age, antidepressant dose (defined daily dose, DDD), age at onset, episode duration, recurrent depression, and education, with a random intercept for participant ID.

## 3. Results

### 3.1. Descriptive Analyses

A total of 117 patients with MDD were included in the statistical analyses. Summaries and test statistics for the univariate comparison analyses of clinical variables among patients with NI (*N* = 84) and those with HI (*N* = 33) are shown in Table 2. The HI group showed a younger age at onset (19.00 [15.00; 22.00] vs. 22.00 [18.00; 27.00]) and more current episodes (21.00 [18.00; 25.00] vs. 24.00 [20.00; 28.50]) than the NI group. Detailed information about all eight SNPs and the results of the cognitive function tasks are shown in Table 1. The results of the PCA of cognitive function showed that most of the variance could be explained by the first two components. Cognitive function was weighted with the corresponding eigenvector coefficients of these two components (Table 3) to construct two new indices (a component of RT and a component of ACC) that explained the different dimensions of cognitive function.

### 3.2. The Interactive Effect Between *EPHX2* and Impulsivity on Depressive Symptoms

**Figure 2:**
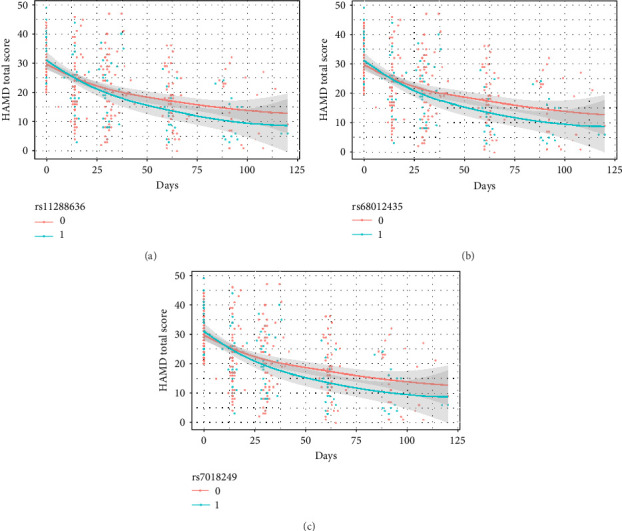
(a–c) The effect of different SNP genotyping on the change of HAMD total score. HAMD, Hamilton Depression Scale-24 items; SNP, single nucleotide polymorphism.

The results of the linear mixed model analyses between SNPs/impulsivity and the prognosis of MDD are presented in Tables 4 and 5, where parameter estimates of different SNPs and their interaction with impulsivity are shown. Block 0 shows an association between impulsivity and the prognosis of MDD, without including SNPs. The interactive effects between SNP and time on the severity of depressive symptoms (measured by the HAMD-24 score) were significantly different and additionally adjusted for the total score of impulsivity (Table 5). At baseline, the HAMD-24 scores were similar between the different SNP genotyping groups (Figure 2). After the first month follow-up, the mean severity for depressive symptoms was lower in those with the minor alleles of rs11288636 (*p*=0.023), rs68012435 (*p*=0.010), and rs7018249 (*p*=0.009).

### 3.3. The Interactive Effect Between *EPHX2* and Impulsivity on the Risk of Suicide

**Figure 3:**
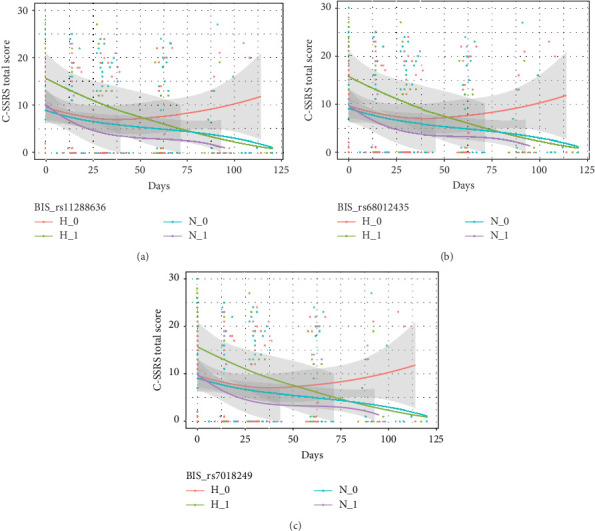
(a–c) The interaction effect between SNP and impulsivity on the change of C-SSRS total score. C-SSRS, Columbia-Suicide Severity Rating Scale; BIS, Barratt Impulsiveness Scale-11; H_1, HI with minor allele; N_0, NI without minor allele; N_1, NI with minor allele; SNP, single nucleotide polymorphism.

Table 4 shows the interactive effects of impulsivity and SNP on the risk of suicide (measured by the C-SSRS score) were significantly different for rs11288636 (*F* = 4.986, *p*=0.017), rs68012435 (*F* = 4.878, *p*=0.020), and rs11288636 (*F* = 4.872, *p*=0.020).  Figure 3 shows the interaction effect obtained by comparing the C-SSRS scores within the HI and NI groups separately between patients with and without the minor alleles of rs11288636, rs68012435, or rs11288636. Post hoc analyses showed no significant interaction effect between SNP genotyping and time in the NI group, whereas, in the HI group, better recovery was found in those with the minor alleles of rs11288636, rs68012435, or rs7018249 (all *p*=0.002). Interactive effects between SNP and time were not found in other SNPs and the change in the risk of suicide.

### 3.4. The Interactive Effect Between *EPHX2* and Impulsivity on Cognitive Function

Table 4 also shows significant interactive effects between impulsivity and SNP on changes in cognitive function (a component of RT). Post hoc analyses showed no significant interaction effect between SNP genotyping and time in the NI group, whereas in the HI group, better recovery was found in those with the minor allele of rs7829267 (*p*=0.029). In addition, no interactive effects were found for the other SNPs, nor was there a change in the severity of depression or cognitive function (a component of the ACC). Interactive effects between SNP and time were not found in other SNPs, nor were they found in changes in cognitive function (both RT and ACC).

### 3.5. Power Analysis

**Figure 4:**
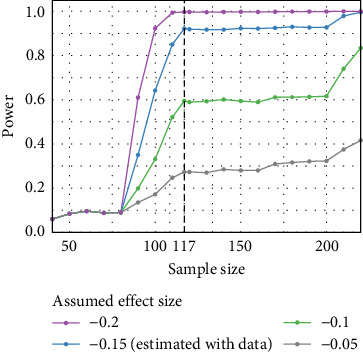
Power analysis of the linear mixed-effect model, with different assumed effect sizes of additional change in the C-SSRS total score over time for group with minor alleles of rs11288636(H_1), relative to the reference group (H_0, with major alleles of rs11288636) within MDD patients with high impulsivity, under the significance level 0.05. C-SSRS, Columbia-Suicide Severity Rating Scale; MDD, major depressive disorder.

Figure 4 presents the results of the power analysis at a significance level of 0.05. The plot shows power curves under different assumed effect sizes of the coefficient of main interest, which is the additional change in the C-SSRS total score over time comparing H_1 versus H_0 (reference). The estimated effect size was −0.15 (Table 4), and we also calculated the power for three other assumed effect sizes (−0.2, −0.1, −0.05). For an estimated effect size of − 0.15, the power surpassed 90% with our sample size of 117 participants (indicated by the vertical dashed line). The analysis indicated that for a small effect size (<−0.1), a sample size of 110 participants was insufficient to provide adequate power. However, for an effect size of −0.15 or larger, a sample size of 110 provided over 80% power. With 117 participants in our study, we achieved a power of over 90%, suggesting that the sample size was sufficient for the analysis. Thus, the sample size of 117 participants was adequate to detect medium to large effects, ensuring the robustness of our model.

## 4. Discussion

Accumulating evidence suggests that *EPHX2* plays an important role in the pathophysiology of MDD. This study explored the effect of *EPHX2* on the prognosis of MDD for the first time. We found that *EPHX2* polymorphisms are associated with MDD prognosis. Additionally, we found that impulsivity could be a critical factor for the change in suicide risk among different *EPHX2* genotypes. To our knowledge, this is the first study to illustrate the association between *EPHX2* expression, impulsivity, and MDD prognosis. Our findings add to the growing evidence that *EPHX2* polymorphisms are potential treatment targets for MDD.

Impulsivity is regarded as a critical factor in the development of suicidal intent and behavior [44] and is considered an endophenotype of suicidal behavior [45]. Impulsivity has been regarded as a mediator in the relationship between depressive symptoms and suicide, including suicidal ideation and behavior [46]. However, recent studies present differing clinical opinions, calling for reconsideration of the link between impulsivity and suicide attempts [47, 48]. No connection was found between trait impulsivity and suicide attempts in a community study [49]. Research also found that suicide attempters do not exhibit higher impulsivity on most measures [48].

In this study, we found that the minor alleles of rs11288636 (8:27489885), rs68012435 (8:27489871), and rs7018249 (8:27514672) had a protective effect on changes in suicide risk among individuals with HI, suggesting that carriers of the above minor allele and HI showed a lower risk of suicide than patients without the minor allele after 3-months of treatment. Rs11288636 and rs68012435 are promoter variants and rs7018249 is an intron variant. Our previous study found that rs11288636 and rs68012435 were in the same haploblock but did not find an association between this haploblock and the risk of suicide/MDD [24]. In this study, rs11288636 and rs68012435 were found to be associated with changes in suicide risk and severity of depressive symptoms, and we suggest that these two SNPs may have cooperative effects through haploblocks. Rs7018249 has been associated with the risk of suicide in a previous study [24]. In the present study, we found an association between the rs7018249 SNP and changes in suicide risk. However, the functions of these three SNPs of *EPHX2* have not been reported. Further studies are required to elucidate the exact functions of these SNPs.

Of particular interest, this result suggests that the previously inconsistent findings regarding the relationship between impulsivity and suicide may be partly explained by genetic factors. Genetic variations, such as the minor alleles identified in our study, might modulate the impact of impulsivity on suicide risk, thereby creating variability in observed clinical outcomes. A shared genetic etiology was found between impulsivity and suicide; therefore, exploring the contribution of genetic factors could help improve suicide prevention [50]. Genetic susceptibility to impulsivity remained a significant predictor for suicide [51]. De Lara et al. [52] demonstrated the possible effect of the genetic variation at the 5-HTT gene on intermediate phenotypes such as impulsive aggressive-behaviors, mediating part of the genetic predisposition to suicide. Huang et al. [53] found that the polymorphism of monoamine oxidase A (MAOA) gene is related to the impulsive traits. These findings underscore the importance of considering genetic contributions when assessing suicide risk in HI individuals. Further research focusing on the interaction between impulsivity and genetic susceptibility could provide more precise insights into suicide prevention strategies, particularly in identifying individuals who are at a reduced risk despite exhibiting HI traits.

Additionally, we found that impulsivity did not affect the role of *EPHX2* in depressive symptoms in MDD. Few studies have focused on the effects of impulsivity on MDD. Some studies found an association between impulsivity and depression [54, 55], whereas others reported conflicting results [27, 56]. Our findings further confirm that impulsivity did not affect the role of *EPHX2* on depressive symptoms in this naturalistic prospective study. In addition, the different effects of impulsivity on depressive symptoms and suicide risk also indicated that suicide was linked to mood disorders but may have an independent physiopathology, similar to our previous study [57].

Impulsivity directly affects changes in cognitive function (a component of RT) without the effect of *EPHX2*. Individuals with HI showed less improvement than those with NI in the RT component during early treatment, whereas they showed better performance after 3-months of treatment. Considering that high performance in the RT component indicates shorter RT in the above domains of cognitive function tasks, our findings suggest that HI is more likely to provide quicker responses during cognitive function assessment.

This is the first study to explore the effects of *EPHX2* on the prognosis of patients with MDD. In previous studies, sEH inhibitors have shown potential as rapid-acting antidepressants. This study further demonstrates that polymorphisms in *EPHX2*, the gene encoding sEH, may influence the prognosis of MDD, including the severity of depressive symptoms, suicide risk, and cognitive function improvement. Future research should further investigate the role of *EPHX2* and sEH in the diagnosis and treatment of MDD, ultimately providing more personalized treatment options for patients. Potential strategies include: (1) tailoring treatment and follow-up plans based on *EPHX2* polymorphisms; and (2) developing new antidepressants targeting *EPHX2*, offering more treatment options for MDD patients. Impulsivity could be a critical factor for the change in suicide risk among different *EPHX2* genotypes. Therefore, clinicians should pay attention to the risk of suicide in MDD with HI and conduct individualized treatments in clinical practice.

## 5. Conclusion

This study is the first to investigate the impact of *EPHX2* on the prognosis of patients with MDD. Our findings suggest that *EPHX2* polymorphisms are associated with the prognosis of MDD, and impulsivity could be a critical factor for the change in suicide risk among different *EPHX2* genotypes. Additionally, impulsivity did not appear to affect the role of *EPHX2* in the modulation of depressive symptom changes in MDD patients.

## 6. Limitations

This study has certain limitations. First, it was conducted at a single center with a small sample size. Second, as a naturalistic prospective study, it had a high dropout rate due to COVID-19. Third, we did not assess changes in impulsivity during the follow-up period. Finally, the cognitive, genetic, and depression subtype (such as seasonal, melancholic, or atypical depression) data were not comprehensive. Future research should include additional information.

## Figures and Tables

**Table 1:** Genotyping and cognitive features at baseline, as well as comparison results without adjusting covariates.

	Total	Normal impulsivity	High impulsivity	*p*-Value
	(*N* = 117)	(*N* = 84)	(*N* = 33)	
SNP genotyping	—	—	—	—
rs11288636	—	—	—	0.680
0 — TTTTTTTT.TTTTTTTT	83 (70.94%)	61 (72.62%)	22 (66.67%)	—
1 — TTTTTTT.TTTTTTTT	34 (29.06%)	23 (27.38%)	11 (33.33%)	—
rs68012435	—	—	—	0.778
0 — CC	82 (70.09%)	60 (71.43%)	22 (66.67%)	—
1 — CG	35 (29.91%)	24 (28.57%)	11 (33.33%)	—
rs7018249	—	—	—	0.878
0 — GG	81 (69.23%)	59 (70.24%)	22 (66.67%)	—
1 — AG	36 (30.77%)	25 (29.76%)	11 (33.33%)	—
rs7829267	—	—	—	0.381
0 — CC	66 (56.41%)	50 (59.52%)	16 (48.48%)	—
1 — TC	51 (43.59%)	34 (40.48%)	17 (51.52%)	—
rs56834178	—	—	—	1.000
0 - CC	75 (64.10%)	54 (64.29%)	21 (63.64%)	—
1 - TC	42 (35.90%)	30 (35.71%)	12 (36.36%)	—
rs9331942	—	—	—	0.594
0 — AA	56 (47.86%)	42 (50.00%)	14 (42.42%)	—
1 — AG	61 (52.14%)	42 (50.00%)	19 (57.58%)	—
rs17466684	—	—	—	0.955
0 — GG	90 (76.92%)	64 (76.19%)	26 (78.79%)	—
1 — AG	27 (23.08%)	20 (23.81%)	7 (21.21%)	—
rs9331949	—	—	—	0.413
0 — TT	69 (58.97%)	52 (61.90%)	17 (51.52%)	—
1 — TC	48 (41.03%)	32 (38.10%)	16 (48.48%)	—
Performance of cognitive function				
ANT_mean ACC	0.98 (0.97; 0.99)	0.99 (0.97; 1.00)	0.98 (0.95; 0.99])	0.048
ANT_mean RT	586.43 (531.34; 652.49)	586.74 (534.73; 657.62)	586.03 (522.28; 652.21)	0.844
ANT_Alerting	18.89 ± 25.40	18.79 ± 26.09	19.13 ± 23.93	0.949
ANT_Orienting	24.08 (3.30; 43.76)	23.98 (3.21; 42.14)	26.38 (3.30; 45.06)	0.613
ANT_executive control	72.59 (47.49; 97.13)	72.82 (45.81; 93.77)	66.50 (49.34; 99.08)	0.617
1back_ACC	0.90 (0.81; 0.95)	0.89 (0.82; 0.96)	0.90 (0.81; 0.94)	0.952
1back_RT	692.75 ± 117.62	694.49 ± 115.29	688.31 ± 125.09	0.799
2back_ACC	0.69 (0.54; 0.79)	0.68 (0.51; 0.78)	0.72 (0.59; 0.82)	0.140
2back_RT	825.63 ± 129.60	815.51 ± 138.13	851.38 ± 102.20	0.179
SST_negative ACC	1.00 (0.96; 1.00)	1.00 (0.96; 1.00)	0.96 (0.92; 1.00)	0.260
SST_positive ACC	0.96 (0.96; 1.00)	1.00 (0.96; 1.00)	0.96 (0.88; 1.00)	0.008
SST_neutral ACC	0.96 (0.96; 1.00)	0.98 (0.96; 1.00)	0.96 (0.88; 1.00)	0.319
SST_suicide ACC	1.00 (0.92; 1.00)	1.00 (0.94; 1.00)	0.96 (0.88; 1.00)	0.019
SST_negative RT	543.95 (480.71; 658.76)	543.19 (474.72; 649.40)	566.70 (500.13; 694.63)	0.349
SST_positive RT	568.20 (486.39; 647.91)	566.05 (486.99; 639.92)	572.58 (471.91; 667.12)	0.567
SST_neutral RT	554.71 (482.96; 646.83)	549.03 (481.09; 634.34)	578.15 (483.52; 750.58)	0.377
SST_suicide RT	545.11 (480.50; 678.59)	541.24 (474.56; 649.97)	596.95 (494.00; 723.26)	0.232

Abbreviations: ACC, accuracy; ANT, attention network test; RT, reaction time; SST, suicide stroop task.

**Table 2:** Demographic information at baseline, as well as comparison results without adjusting covariates.

	Total	Normal impulsivity	High impulsivity	*p*-Value
	(*N* = 117)	(*N* = 84)	(*N* = 33)	
Age (years)	24.00 (21.00; 29.00)	25.00 (21.00; 29.00)	23.00 (20.00; 30.00)	0.132
Gender	—	—	—	0.108
Male	45 (38.46%)	28 (33.33%)	17 (51.52%)	—
Female	72 (61.54%)	56 (66.67%)	16 (48.48%)	—
Marriage status	—	—	—	0.441
Single	86 (73.50%)	61 (72.62%)	25 (75.76%)	—
Married	27 (23.08%)	21 (25.00%)	6 (18.18%)	—
Divorced	4 (3.42%)	2 (2.38%)	2 (6.06%)	—
Educational level	—	—	—	0.789
Above high school	74 (63.25%)	52 (61.90%)	22 (66.67%)	—
Other	43 (36.75%)	32 (38.10%)	11 (33.33%)	—
BMI	20.69 (18.69; 23.63)	20.32 (18.73; 22.59)	21.61 (18.55; 24.35)	0.332
Age at onset (years)	21.00 (17.00; 25.00)	22.00 (18.00; 27.00)	19.00 (15.00; 22.00)	0.011
Age at current episode (years)	23.00 (19.00; 27.00)	24.00 (20.00; 28.50)	21.00 (18.00; 25.00)	0.025
Duration of current episode (weeks)	20.00 (5.00; 96.00)	20.00 (5.50; 64.00)	22.00 (5.00; 240.00)	0.333
Recurrent major depressive episode	—	—	—	0.288
No	71 (60.68%)	54 (64.29%)	17 (51.52%)	—
Yes	46 (39.32%)	30 (35.71%)	16 (48.48%)	—
Family history of psychiatric disorder	—	—	—	0.468
No	92 (78.63%)	68 (80.95%)	24 (72.73%)	—
Yes	25 (21.37%)	16 (19.05%)	9 (27.27%)	—
HAMD_total score	31.68 ± 6.27	31.10 ± 6.41	33.18 ± 5.72	0.106
C-SSRS_total score	14.00 (0.00; 19.00)	13.50 (0.00; 19.00)	15.00 (1.00; 20.00)	0.198
BIS_total score	67.72 ± 7.73	64.04 ± 4.93	77.09 ± 5.23	<0.001
BIS_cognitive impulsivity	16.37 ± 3.02	15.60 ± 2.79	18.33 ± 2.69	<0.001
BIS_motor impulsivity	20.44 ± 3.85	19.14 ± 2.93	23.73 ± 3.99	<0.001
BIS_non planning impulsivity	28.68 ± 3.93	27.27 ± 3.31	32.24 ± 3.06	<0.001

Abbreviations: BIS, Barratt Impulsiveness Scale-11; C-SSRS, Columbia-Suicide Severity Rating Scale; HAMD, Hamilton Depression Scale-24 items.

**Table 3:** Component Matrix of each principal component of cognitive function.

Component Matrix	Principal component 1	Principal component 2
ANT_mean_ACC	0.385	**0.536**
ANT_mean_RT	**−0.748**	0.264
ANT_Alerting	0.105	−0.031
ANT_Orienting	0.283	0.08
ANT_Executive control	−0.222	−0.119
1back_ACC	0.510	0.343
1back_RT	**−0.683**	0.247
2back_ACC	0.561	0.215
2back_RT	−0.403	0.316
SST_negative ACC	0.318	**0.710**
SST_positive ACC	0.317	**0.729**
SST_neutral ACC	0.327	**0.716**
SST_suicide ACC	0.393	**0.660**
SST_negative RT	**−0.890**	0.297
SST_positive RT	**−0.893**	0.284
SST_neutral RT	**−0.896**	0.281
SST_suicide RT	**−0.876**	0.268

*Note*: The bold values indicate the highest loading for each variable across the two principal components, and they should be shown in normal font style for consistency.

Abbreviations: ACC, accuracy; ANT, attention network test; RT, reaction time; SST, suicide stroop task.

**Table 4:** The interactive effect between impulsivity and different genotyping of SNP on the prognosis of MDD.

Independent Variables	Dependent variables											
	HAMD total score			C-SSRS total score			Component of RT			Component of ACC		
	Estimate	*p*-Value	Adjusted *p*-Value*⁣*^*∗*^	Estimate	*p*-Value	Adjusted *p*-Value*⁣*^*∗*^	Estimate	*p*-Value	Adjusted *p*-Value*⁣*^*∗*^	Estimate	*p*-Value	Adjusted *p*-Value*⁣*^*∗*^
Block0												
Days*⁣*^*∗*^high impulsivity	−0.005	0.841	—	−0.015	0.542	—	0.016	0.003	—	−0.002	0.693	—
Block1												
Days impulsivity_rs11288636	—	—	—	—	—	—	—	—	—	—	—	—
Days (refer: H_0)	−0.133	<0.001	<0.001	−0.005	0.836	1.000	0.026	<0.001	<0.001	−0.012	0.020	0.156
Days H_1	−0.126	0.001	0.011	−0.146	<0.001	0.002	0.017	0.036	0.289	0.015	0.055	0.442
Days N_0	−0.037	0.223	1.000	−0.037	0.217	1.000	−0.009	0.166	1.000	0.006	0.317	1.000
Days N_1	−0.073	0.071	0.568	−0.058	0.149	1.000	−0.012	0.156	1.000	0.014	0.090	0.717
Block2												
Days impulsivity_rs68012435	—	—	—	—	—	—	—	—	—	—	—	—
Days (refer: H_0)	−0.133	<0.001	<0.001	−0.005	0.842	1.000	0.026	<0.001	<0.001	−0.012	0.020	0.156
Days H_1	−0.126	0.001	0.011	−0.146	<0.001	0.002	0.017	0.037	0.295	0.015	0.056	0.444
Days N_0	−0.034	0.267	1.000	−0.040	0.181	1.000	−0.009	0.152	1.000	0.006	0.312	1.000
Days N_1	−0.083	0.038	0.308	−0.047	0.237	1.000	−0.010	0.201	1.000	0.013	0.096	0.767
Block3												
Days impulsivity_rs7018249	—	—	—	—	—	—	—	—	—	—	—	—
Days (refer: H_0)	−0.133	<0.001	<0.001	−0.005	0.839	1.000	0.026	<0.001	<0.001	−0.012	0.020	0.156
Days H_1	−0.126	0.001	0.011	−0.146	<0.001	0.002	0.017	0.037	0.296	0.015	0.055	0.443
Days N_0	−0.034	0.267	1.000	−0.040	0.181	1.000	−0.009	0.152	1.000	0.006	0.316	1.000
Days N_1	−0.083	0.037	0.296	−0.047	0.231	1.000	−0.010	0.200	1.000	0.013	0.096	0.770
Block4												
Days impulsivity_rs7829267	—	—	—	—	—	—	—	—	—	—	—	—
Days (refer: H_0)	−0.137	<0.001	< 0.001	−0.002	0.958	1.000	0.020	<0.001	0.006	−0.011	0.072	0.574
Days H_1	−0.085	0.029	0.231	−0.113	0.003	0.028	0.023	0.004	0.029	0.009	0.249	1.000
Days N_0	−0.037	0.298	1.000	−0.048	0.170	1.000	0.000	0.992	1.000	0.004	0.580	1.000
Days N_1	−0.044	0.224	1.000	−0.040	0.263	1.000	−0.008	0.294	1.000	0.010	0.178	1.000
Block5												
Days impulsivity_rs56834178	—	—	—	—	—	—	—	—	—	—	—	—
Days (refer: H_0)	−0.176	<0.001	<0.001	−0.072	0.004	0.028	0.037	<0.001	<0.001	−0.003	0.536	1.000
Days H_1	−0.026	0.523	1.000	0.020	0.628	1.000	−0.015	0.070	0.563	−0.009	0.299	1.000
Days N_0	−0.003	0.929	1.000	0.031	0.326	1.000	−0.021	0.001	0.010	−0.003	0.683	1.000
Days N_1	−0.006	0.862	1.000	0.009	0.800	1.000	−0.021	0.003	0.026	0.001	0.828	1.000
Block6												
Days impulsivity_rs9331942	—	—	—	—	—	—	—	—	—	—	—	—
Days (refer: H_0)	−0.171	<0.001	<0.001	−0.071	0.025	0.198	0.035	<0.001	<0.001	−0.003	0.643	1.000
Days H_1	−0.022	0.584	1.000	0.010	0.804	1.000	−0.005	0.572	1.000	−0.005	0.555	1.000
Days N_0	0.002	0.958	1.000	0.033	0.397	1.000	−0.018	0.023	0.184	0.000	0.951	1.000
Days N_1	−0.016	0.680	1.000	0.011	0.773	1.000	−0.019	0.015	0.122	−0.001	0.889	1.000
Block7												
Days impulsivity_rs17466684	—	—	—	—	—	—	—	—	—	—	—	—
Days (refer: H_0)	−0.184	<0.001	<0.001	−0.074	0.001	0.008	0.035	<0.001	<0.001	−0.004	0.342	1.000
Days H_1	−0.005	0.908	1.000	0.038	0.401	1.000	−0.011	0.236	1.000	−0.008	0.422	1.000
Days N_0	0.000	0.998	1.000	0.006	0.821	1.000	−0.020	0.001	0.006	−0.001	0.882	1.000
Days N_1	0.014	0.708	1.000	0.078	0.038	0.307	−0.013	0.093	0.742	0.004	0.580	1.000
Block8												
Days impulsivity_rs9331949	—	—	—	—	—	—	—	—	—	—	—	—
Days (refer: H_0)	−0.153	<0.001	<0.001	−0.060	0.035	0.279	0.031	<0.001	<0.001	−0.003	0.558	1.000
Days H_1	−0.062	0.114	0.913	−0.008	0.830	1.000	0.003	0.662	1.000	−0.005	0.522	1.000
Days N_0	−0.031	0.386	1.000	0.014	0.684	1.000	−0.015	0.039	0.314	−0.002	0.782	1.000
Days N_1	−0.024	0.524	1.000	0.006	0.881	1.000	−0.013	0.094	0.752	0.001	0.876	1.000

Abbreviations: ACC, accuracy; C-SSRS, Columbia-Suicide Severity Rating Scale; H_1, HI with minor allele; HAMD, Hamilton Depression Scale-24 items; N_0, NI without minor allele; N_1, NI with minor allele; RT, reaction time.

*⁣* ^*∗*^Linear mixed-effect model adjusted for age, sex, age at onset, age at current episode, educational level, recurrent episode or not, and the DDD of the antidepressant. Bonferroni correction were used for multiple comparisons.

**Table 5:** The interactive effect between different genotyping of SNP and time on the prognosis of MDD.

Independent variables	Dependent variables											
	HAMD total score			C-SSRS total score			Component of RT			Component of ACC		
	Estimate	*p*-Value	Adjusted *p*-Value*⁣*^*∗*^	Estimate	*p*-Value	Adjusted *p*-Value*⁣*^*∗*^	Estimate	*p*-Value	Adjusted *p*-Value*⁣*^*∗*^	Estimate	*p*-Value	Adjusted *p*-Value*⁣*^*∗*^
Block1												
Days	−0.159	<0.001	<0.001	—	—	—	—	—	—	−0.008	0.012	0.098
rs11288636	1.421	0.382	1.000	—	—	—	—	—	—	−0.441	0.201	1.000
BIS total score	0.239	0.011	0.090	—	—	—	—	—	—	−0.009	0.651	1.000
Days*⁣*^*∗*^rs11288636	−0.078	0.003	0.023	—	—	—	—	—	—	0.011	0.041	0.329
Block2												
Days	−0.157	<0.001	<0.001	—	—	—	—	—	—	−0.008	0.013	0.104
rs68012435	1.301	0.419	1.000	—	—	—	—	—	—	−0.375	0.273	1.000
BIS total score	0.239	0.011	0.087	—	—	—	—	—	—	−0.009	0.643	1.000
Days rs68012435	−0.084	0.001	0.010	—	—	—	—	—	—	0.010	0.047	0.374
Block3												
Days	−0.157	<0.001	<0.001	—	—	—	—	—	—	−0.008	0.013	0.101
rs7018249	1.094	0.492	1.000	—	—	—	—	—	—	−0.425	0.209	1.000
BIS total score	0.241	0.010	0.080	—	—	—	—	—	—	−0.010	0.613	1.000
Days rs7018249	−0.084	0.001	0.009	—	—	—	—	—	—	0.010	0.045	0.356
Block4												
Days	−0.163	<0.001	<0.001	−0.036	0.052	0.418	—	—	—	−0.008	0.030	0.236
rs7829267	−0.471	0.757	1.000	1.053	0.488	1.000	—	—	—	−0.433	0.178	1.000
BIS total score	0.232	0.014	0.108	0.123	0.187	1.000	—	—	—	−0.006	0.741	1.000
Days rs7829267	−0.037	0.123	0.985	−0.040	0.094	0.748	—	—	—	0.007	0.166	1.000
Block5												
Days	−0.178	<0.001	<0.001	−0.053	0.001	0.012	—	—	—	−0.004	0.187	1.000
rs56834178	0.140	0.929	1.000	−0.201	0.897	1.000	—	—	—	−0.201	0.543	1.000
BIS total score	0.222	0.020	0.158	0.119	0.203	1.000	—	—	—	−0.011	0.593	1.000
Days rs56834178	−0.012	0.618	1.000	−0.008	0.740	1.000	—	—	—	0.000	0.920	1.000
Block6												
Days	−0.173	<0.001	<0.001	−0.049	0.012	0.097	0.024	<0.001	<0.001	−0.003	0.412	1.000
rs9331942	−0.631	0.670	1.000	0.093	0.950	1.000	0.025	0.952	1.000	−0.270	0.385	1.000
BIS total score	0.221	0.019	0.150	0.122	0.186	1.000	−0.018	0.510	1.000	−0.009	0.643	1.000
Days rs9331942	−0.017	0.497	1.000	−0.012	0.612	1.000	−0.001	0.806	1.000	−0.002	0.641	1.000
Block7												
Days	−0.185	<0.001	<0.001	−0.071	<0.001	<0.001	0.024	<0.001	<0.001	−0.005	0.108	0.864
rs17466684	−1.698	0.351	1.000	−2.308	0.203	1.000	−0.301	0.562	1.000	0.356	0.355	1.000
BIS total score	0.217	0.021	0.167	0.120	0.197	1.000	−0.020	0.477	1.000	−0.006	0.750	1.000
Days rs17466684	0.006	0.820	1.000	0.060	0.031	0.246	0.000	0.973	1.000	0.001	0.858	1.000
Block8												
Days	−0.174	<0.001	<0.001	−0.052	0.004	0.031	0.022	<0.001	<0.001	−0.005	0.196	1.000
rs9331949	0.375	0.803	1.000	0.397	0.791	1.000	−0.306	0.471	1.000	0.109	0.731	1.000
BIS total score	0.223	0.018	0.146	0.124	0.182	1.000	−0.019	0.501	1.000	−0.008	0.690	1.000
Days rs9331949	−0.019	0.426	1.000	−0.011	0.657	1.000	0.004	0.456	1.000	0.000	0.966	1.000

Abbreviations: ACC, accuracy; C-SSRS, Columbia-Suicide Severity Rating Scale; H_1, HI with minor allele; HAMD, Hamilton Depression Scale-24 items; N_0, NI without minor allele; N_1, NI with minor allele; RT, reaction time.

*⁣* ^*∗*^Linear mixed-effect model adjusted for age, sex, age at onset, age at current episode, educational level, recurrent episode or not, the DDD of the antidepressant and BIS-11 total score. Bonferroni correction were used for multiple comparisons.

## Data Availability

The data that support the findings of this study are available from the corresponding author upon reasonable request.
